# Prognostic impact of translocation t(14;16) in multiple myeloma according to the presence of additional genetic lesions

**DOI:** 10.1038/s41408-023-00933-4

**Published:** 2023-10-26

**Authors:** Anaïs Schavgoulidze, Aurore Perrot, Titouan Cazaubiel, Xavier Leleu, Lydia Montes, Caroline Jacquet, Karim Belhadj, Sabine Brechignac, Laurent Frenzel, Thomas Chalopin, Philippe Rey, Jean-Marc Schiano de Collela, Mamoun Dib, Denis Caillot, Margaret Macro, Jean Fontan, Laure Buisson, Luka Pavageau, Murielle Roussel, Salomon Manier, Mohamad Mohty, Ludovic Martinet, Hervé Avet-Loiseau, Jill Corre

**Affiliations:** 1 Unit for Genomics in Myeloma, University Hospital IUCT-Oncopole, Toulouse, France; 2 Cancer Research Center of Toulouse (CRCT), Institut National de la Santé et de la Recherche Médicale (INSERM), Centre National de la Recherche Scientifique (CNRS), Université Toulouse III-Paul Sabatier (UPS), Toulouse, France; 3 Hematology Department, IUCT-Oncopole, Toulouse, France; 4 Hematology Department, University Hospital, Bordeaux, France; 5 Hematology Department, University Hospital, Poitiers, France; 6 Hematology Department, University Hospital, Amiens, France; 7 Hematology Department, University Hospital, Nancy, France; 8 Hematology Department, University Hospital, Créteil, France; 9 Hematology Department, University Hospital, Bobigny, France; 10 Hematology Department, Necker University Hospital, Paris, France; 11 Hematology Department, University Hospital, Tours, France; 12 Hematology Department, Centre Léon Bérard, Lyon, France; 13 Hematology Department, Institut Paoli Calmettes, Marseille, France; 14 Hematology Department, University Hospital, Angers, France; 15 Hematology Department, Institut de Cancérologie de Bourgogne, Dijon, France; 16 Hematology Department, University Hospital, Caen, France; 17 Hematology Department, University Hospital, Besançon, France; 18 Hematology Department, University Hospital, Limoges, France; 19 Hematology Department, University Hospital, Lille, France; 20 Hematology Department, Saint-Antoine University Hospital, Paris, France

**Keywords:** Risk factors, Genetics research, Myeloma

Patients with multiple myeloma (MM) have experienced a markedly improved survival over the past 25 years, thanks in part to the availability of new drugs [1]. However, a subgroup of patients known as high-risk patients continues to represent an unmet medical need, with an overall survival of less than three years [2]. In the context where we aim to design clinical trials dedicated to high-risk myeloma patients, these patients must be accurately identified from diagnosis. Given the major prognostic role of cytogenetics in myeloma, the Revised International Staging System has included three cytogenetic abnormalities (CA) in 2015: the deletion 17p and the translocations t(4;14) and t(14;16), along with LDH level [3]. The t(14;16) is also considered as a high-risk factor in the m-SMART risk stratification [4]. Whereas le prognostic role of t(4;14) and deletion 17p is widely accepted, the independent prognostic impact of t(14;16) has been a matter of debate [5–8]. This rare translocation (around 3.5% of MM patients) involves the *IGH* locus and the oncogene c-MAF, whose overexpression has been shown to mediate innate resistance to proteasome inhibitors [9]. In addition, t(14;16) MM cells clearly display a much higher mutations number and a higher APOBEC-related mutational process than non-t(14;16) MM cells [10, 11].

**Fig. 1:**
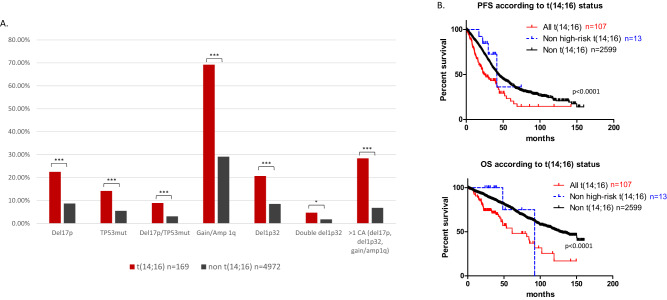
Genetic profile and clinical outcome of NDMM patients with t(14;16). **A** Frequencies of prognostic abnormalities according to t(14;16) status. The black bars correspond to 4972 newly diagnosed patients without t(14;16), the red bars to 169 newly diagnosed patients with t(14;16). Categorical data are presented as percentages and compared using a chi-square test. ***P < 0.0001, *P = 0.013. **B** Kaplan–Meier progression-free and overall survival of patients with NDMM according to cytogenetics. The red curve corresponds to all patients with t(14;16), the blue dotted curve to patients with t(14;16) but without deletion 17p, TP53 mutation, gain/amp 1q, deletion 1p32, the black curve to patients without t(14;16). *P* values are determined by the log-rank test comparison.

In this study, we performed targeted next-generation sequencing as previously described [12] on bone marrow CD138-positive sorted cells, obtained in 5141 newly diagnosed myeloma (NDMM) patients. Raw data are available on demand. The Toulouse Ethics Committee approved the study and informed consent was obtained for all included patients. We found a t(14;16) in 169/5141 newly diagnosed patients (3,3%), which was the expected proportion. We observed that t(14;16) was highly associated to other high-risk abnormalities (Fig. 1A). Indeed, 69.2% of patients with t(14;16) also displayed gain/amp 1q vs 29.1% for patients without t(14;16) (*P* < 0.0001, chi-square test). Patients with t(14;16) also displayed significantly more deletion 1p32 (20.7% vs 8.5%, *P* < 0.0001), including more biallelic deletion 1p32 (4.7% vs 1.8%, *P* = 0.013) recently described as a very high-risk entity [12]. A deletion 17p was found in 22.5% of patients with t(14;16), vs 8.7% in patients without t(14;16) (*P* < 0.0001). We found 8.9% of biallelic inactivation of *TP53* in the t(14;16) subgroup, vs 3.1% in the non t(14;16) subgroup (*P* < 0.0001). Globally, patients with t(14;16) displayed a *TP53* mutation in 14.2% of cases, vs 5.5% for non-t (14;16) patients (*P* < 0.0001). Finally, we compared the proportion of patients with at least two cytogenetic abnormalities among deletion 17p, gain/amp1q, and deletion 1p32. This proportion was 28.4% for patients with t(14;16) vs 6.8% for patients without t(14;16) (*p* < 0.0001). Only 30/169 t(14;16) patients displayed none abnormality among deletion 17p, *TP53* mutation, and chromosome 1 abnormalities.

It has been suggested that patients with t(14;16) were more at risk to have renal impairment [7], that could lead to an underrepresentation of this cytogenetic abnormality in clinical trials. However in this cohort of NDMM patients (not included in clinical trials), those with t(14;16) did not significantly develop more renal failure than other patients (15.4% vs 12.1%, *P* = 0.3).

Clinical data were available for 2706 NDMM patients (median age 64 years (27–92)) diagnosed between January 2010 and January 2022, followed up for ≥18 months or having died or progressed within 18 months. The median follow-up was 34.6 months. Almost half of the patients (1335) were treated with intensive therapy (classical triplet induction + autologous stem cell transplant, except for 86 patients who received bortezomib-dexamethasone induction, and 194 patients who had anti-CD38 based quadruplet). Half of the non-transplant-eligible patients received lenalidomide + bortezomib + dexamethasone, or lenalidomide + dexamethasone; the other half received an anti-CD38 combined with Lenalidomide and dexamethasone (360 patients) or one of the following combination: bortezomib + cyclophosphamide + dexamethasone or melphalan + prednisone + bortezomib or melphalan + prednisone + thalidomide. Rates of progression-free survival (PFS) and overall survival (OS) were estimated by the Kaplan–Meier method. Tests were two-sided and *P*<0.05 were considered significant. The median PFS was 24.3 months for the 107 patients with t(14;16) vs 43.9 months for patients without t(14;16) (*P* < 0.0001). The median OS was 61.3 months for the patients with t(14;16) vs 128.8 months for patients without it (*P* < 0.0001) (Fig. 1B). Given the high association we found between t(14;16) and deletion 17p, *TP53* mutation, gain/amp 1q, and deletion 1p32, we then focused on t(14;16) patients without any of these lesions. PFS and OS were not more significantly shorter than patients without t(14;16) (41.5 and 92.3 months, respectively) (Fig. 1B). Even if the number is very small (only 13 patients from this clinically annotated patient cohort), these data strongly suggest that t(14;16) with no other high-risk lesion may not be considered as a poor factor in MM. Of note, the Revised 2-ISS [13] takes into account 1q gain as a high-risk CA, but not t(14;16). In 2019, the IFM proposed a weighted cytogenetic score [14] notably based on deletion 17p, t(4;14), 1q gain and deletion 1p32, but t(14;16) was not retained in the prognostic model. Of note, in this system, only a deletion 17p was able to confer a poor prognostic by itself, the other lesions had to be combined with each other.

A quite similar situation than for t(14;16) was observed a few years ago with deletion 13q, which has been a quite good surrogate marker for MM high-risk disease, but not an independent factor, since patients with deletion 13q but without deletion 17p nor t(4;14) had similar outcome than patients without deletion 13q [15]. With t(14;16), it is more difficult to address because it is a much rare event whereas deletion 13q is present in about half of NDMM patients.

**Fig. 2:**
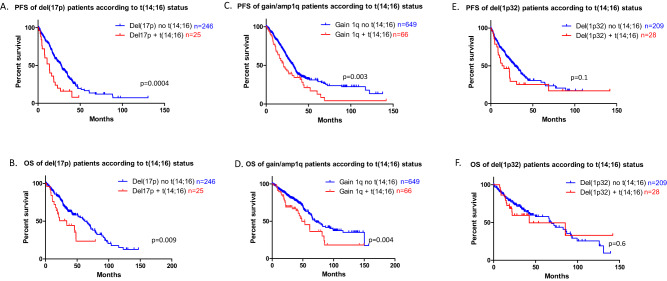
Clinical outcome of NDMM patients with deletion 17p or gain 1q or deletion 1p32, according to t(14;16) status. Kaplan–Meier survival of NDMM patients according to cytogenetics. **A** (PFS) and **B** (OS): the red curve corresponds to all patients with deletion 17p without t(14;16), the blue curve to patients with both deletion 17p and t(14;16). **C** (PFS) and **D** (OS): the red curve corresponds to all patients with gain/amplification 1q without t(14;16), the blue curve to patients with both gain 1q and t(14;16). **E** (PFS) and **F** (OS): the red curve corresponds to all patients with deletion 1p32 without t(14;16), the blue curve to patients with both deletion 1p32 and t(14;16). *P* values are determined by the log-rank test comparison.

At this stage, the question was whether t(14;16) assessment is still useful for defining risk in MM. To address this issue, we asked if the presence of a t(14;16) worsen the prognosis of patients presenting a cytogenetic lesion associated to a shorter survival at diagnosis (Fig. 2). For patients with a deletion 17p, the PFS and OS were clearly shorter for those displaying a t(14;16) (13.5 vs 27.4 months, *P* < 0.0001, and 33.5 vs 62.4 months, *P* = 0.009, respectively). Although to a lesser extent, the same observation was done for patients with gain/amplification 1q, (PFS 20.5 vs 31.8 months, *P* = 0.003 and OS 48.2 vs 69.9 months, *P* = 0.004). Only a tendency was observed for with a deletion 1p32 (PFS 14.6 vs 28.5 months, *P* = 0.11, and OS 42.4 vs 69.0 months, *P* = 0.26). These data suggest that the interaction of a t(14;16) with a high-risk cytogenetic lesion can lead to a particularly aggressive disease. The biological mechanism underlying this observation needs to be elucidated.

Our study has several limitations: it is retrospective, treatments are not homogenous, and the cohort size of t(14;16) patients remains small, especially since, by definition, we focused on patients without deletion 17p, *TP53* mutation, gain/amp 1q and deletion 1p32.

In conclusion, two third of patients with t(14;16) also display a gain/amp 1q. They also have two to three times more deletion 17p, *TP53* mutations and deletion 1p32 than other patients, and almost one-third display at least two of these abnormalities. The t(14;16) has not any prognostic impact if isolated (but numbers are very small). In contrast, its interaction with another prognostic lesion can lead to an aggressive disease. We state that only t(14;16) associated to other high-risk abnormalities should be considered as a high-risk disease in MM.
